# Novel immunotherapeutic effects of topically administered ripasudil (K-115) on corneal allograft survival

**DOI:** 10.1038/s41598-020-76882-w

**Published:** 2020-11-13

**Authors:** Takenori Inomata, Keiichi Fujimoto, Yuichi Okumura, Jun Zhu, Kenta Fujio, Hurramhon Shokirova, Maria Miura, Mikiko Okano, Toshinari Funaki, Jaemyoung Sung, Naoko Negishi, Akira Murakami

**Affiliations:** 1 Department of Ophthalmology, Juntendo University Graduate School of Medicine, 3-1-3 Hongo, Bunkyo-ku, Tokyo, 113-0033 Japan; 2 Department of Ophthalmology, Juntendo University Faculty of Medicine, Tokyo, Japan; 3 Department of Strategic Operating Room Management and Improvement, Juntendo University Graduate School of Medicine, Tokyo, Japan; 4 Department of Hospital Administration, Juntendo University Graduate School of Medicine, Tokyo, Japan; 5 Department of Digital Medicine, Juntendo University Graduate School of Medicine, Tokyo, Japan; 6 Department of Ophthalmology, Japanese Red Cross Medical Center, Tokyo, Japan; 7 Morsani College of Medicine, University of South Florida, Tampa, FL USA; 8 Atopy (Allergic) Research Center, Juntendo University Graduate School of Medicine, Tokyo, Japan; 9 Department of Indoor Environment Neurophysiology Research, Juntendo University Graduate School of Medicine, Tokyo, Japan

**Keywords:** Eye diseases, Corneal diseases, Transplant immunology

## Abstract

Corneal allograft survival is mediated by the variety of immunological reactions and wound healing process. Our aim was to explore the effects of topical administration of ripasudil, a selective Rho-associated coiled-coil protein kinase inhibitor, on corneal allograft survival. Ripasudil was administered to mice thrice a day after allogeneic corneal transplantation. Corneal graft survival, opacity, neovascularization, re-epithelization, immune cell infiltration, and mRNA levels of angiogenic and pro-inflammatory factors in the grafted cornea and draining lymph nodes (dLNs) were evaluated with slit-lamp microscopy, immunohistochemistry, flow cytometry, and polymerase chain reaction. Graft survival was significantly prolonged with lower graft opacity and neovascularization scores in 0.4% and 2.0% ripasudil-treated groups, and mRNA levels of angiogenic and pro-inflammatory factors in ripasudil-treated grafted corneas were reduced. Moreover, 0.4% and 2.0% ripasudil reduced CD45^+^-infiltrated leukocyte frequency, *Cd11b* and *Cd11c* mRNA levels, and the frequencies of mature dendritic cells, IFNγ-, and IL-17- producing CD4^+^T cells in the dLNs of recipients. Re-epithelization rate of the grafted cornea was significantly higher in the 0.4% and 2.0% ripasudil groups than in the control. Topically applied ripasudil prolonged graft survival by downregulating neovascularization and inflammation factors, while promoting corneal re-epithelization, suggesting that ripasudil may be useful for suppressing immunological rejection in corneal transplantation.

## Introduction

The cornea is the most commonly transplanted tissue worldwide^1^, and corneal transplantation is associated with high success rates owing to its immune privilege^2^. However, host factors involved in inflammation and neovascularization lead to high rejection rates even with the use of topical steroids^3^. These effects are observed despite treatment with high doses of non-specific immunosuppressive agents, thus often preventing long-term graft survival combined with manifestation of severe side effects, including cataracts, glaucoma, and opportunistic infections^4,5^.

The cornea is an avascular, transparent dome layer tissue that acts as a mechanical barrier and contributes to two-thirds of the refractive power of the eye^6^. Corneal neovascularization (CNV) is associated with several etiologies, including improper use of contact lens, corneal infections, chemical burns, ocular surface inflammation, trauma, limbal stem cell deficiency, and post-corneal transplantation^6^, and is estimated to affect 1.4 million individuals annually^7^. CNV decreases visual acuity with higher-order aberrations and corneal edema, contributing to a worse prognosis of corneal transplantation, accompanied by increased ocular surface inflammation and immunological responses^8^. Furthermore, CNV causes blindness in approximately 7 million individuals worldwide^9^.

CNV affects wound healing and immunological reactions after corneal transplantation by transporting immune cells into and out of the local site^10^. Therefore, suppression of CNV and control of immune cells are required for reducing the rates of rejection after corneal transplantation. Anti-vascular endothelial growth factor (VEGF) is widely used for preventing neovascularization, as VEGF is the primary regulator of human angiogenesis and promotes vascular endothelial cell proliferation, migration, and tube formation^6^.

Despite several studies reporting the beneficial effects of anti-angiogenic therapy for CNV^11–13^, eye drops with anti-angiogenic properties are not yet available in the clinical setting. Therefore, the development of anti-angiogenic eye drops is an unmet medical requirement, as CNV causes not only immune rejection in corneal transplantation but also vision loss due to higher-order aberrations and edema.

Rho-associated coiled-coil-containing protein kinase (ROCK), a target of the small-molecule GTP-binding protein Ras homolog family member A (Rho-A)^14,15^, exists as two isoforms: ROCK1 and ROCK2. The Rho-A/ROCK pathway, along with various cytokines such as VEGF, is associated with angiogenesis^16,17^. The ROCK inhibitor ripasudil hydrochloride hydrate (K-115) selectively inhibits both ROCK1 and ROCK2, and ripasudil (0.4%) has been approved for glaucoma treatment in Japan^18^. Ripasudil can be used as a potential therapeutic eye drop for retinal hypoxic neovascular diseases^19,20^; however, the effects of ripasudil on corneal allograft survival are not known.

Hence, in this study, we evaluated the immunotherapeutic potential of topically administered ripasudil for corneal allograft survival using a murine corneal transplantation model. The present results may provide a foundation for the clinical application of topical ripasudil in the regulation of corneal angiogenesis to improve the prognosis of corneal transplant recipients.

## Results

### Topical administration of ripasudil inhibited graft cornea angiogenesis and lymphangiogenesis

**Figure 1:**
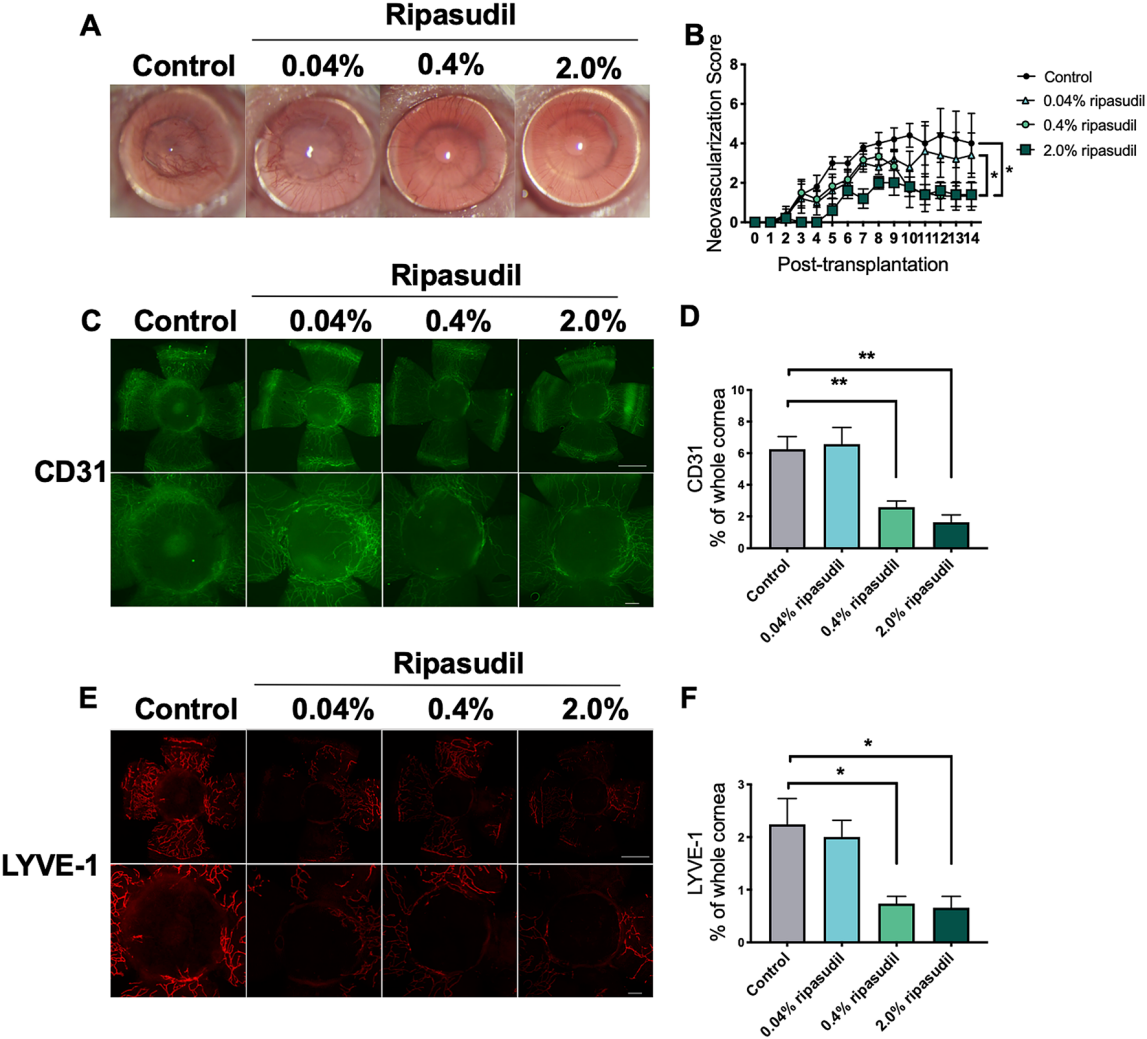
Topical administration of ripasudil suppressed neovascularization and lymphangiogenesis in murine corneal transplants. (**A**) Representative slit-lamp images showing grafted corneas on day 14 post-transplantation (magnification × 25). (**B**) Neovascularization scores of grafted corneas 14 days after transplantation (two-way ANOVA, n = 5 per group; *p < 0.01). (**C**) Representative immunohistochemical images of CD31 staining of grafted corneas on day 14 post-transplantation. Upper row: whole grafted cornea. Bottom row: high magnification showing center of grafted cornea. (**D**) Percentages of the corneal area covered with blood vessels (CD31^+^) in the ripasudil and control groups (one-way ANOVA, n = 5 per group; *p < 0.01). (**E**) Representative immunohistochemical images of lymphatic vessel endothelial hyaluronan receptor-1 (LYVE-1) staining of grafted corneas on day 14 post-transplantation. Upper row: whole grafted cornea. Bottom row: high magnification showing center of grafted cornea. (**F**) Percentages of the corneal area covered with lymphatic vessels (LYVE-1^+^) in the ripasudil and control groups (one-way ANOVA, n = 5 per group; *p < 0.05). The area of the whole grafted cornea covered by blood or lymphatic vessels was analyzed using ImageJ version 1.53a (National Institutes of Health, Bethesda, MD, USA; available at https://rsb.info.nih.gov/ij/index.html)^57,58^. Scale bar, 1000 μm. *ANOVA* analysis of variance.

Figure 1A shows a representative image of the grafted cornea at day 14 post-transplantation. Topical administration of 0.4% and 2.0% ripasudil significantly reduced the CNV score compared to that in the control group on day 14 post-transplantation (Fig. 1B *p* = 0.022 and *p* = 0.037, respectively). Figure 1C shows the results of immunofluorescence staining for blood (CD31^+^) in the grafted corneas. Upper row shows the whole grafted cornea. Bottom row shows the center of the grafted cornea at higher magnification. Quantification of the vascularized area in corneal whole mounts revealed that topical administration of 0.4% and 2.0% ripasudil significantly reduced the de novo generation of CD31^+^ blood vessels (*p* = 1.000, 0.008, and 0.001 for 0.04%, 0.4%, 2.0% ripasudil versus control, respectively; n = 5, Fig. 1D). Figure 1E shows the results of immunofluorescence staining for lymphatic vessels (lymphatic vessel endothelial hyaluronan receptor-1 (LYVE-1)^+^) in the grafted corneas. Upper row shows the whole grafted cornea. Bottom row shows the center of the grafted cornea at higher magnification. Quantification of the vascularized area in corneal whole mounts revealed that topical administration of 0.4% and 2.0% ripasudil significantly reduced the de novo generation of LYVE-1^+^ lymphatic vessels (*p* = 1.000, 0.012, and 0.009 vs. control, respectively; n = 5, Fig. 1F).

### Topical administration of ripasudil downregulated angiogenic and lymphangiogenic factors in grafted corneas

**Figure 2:**
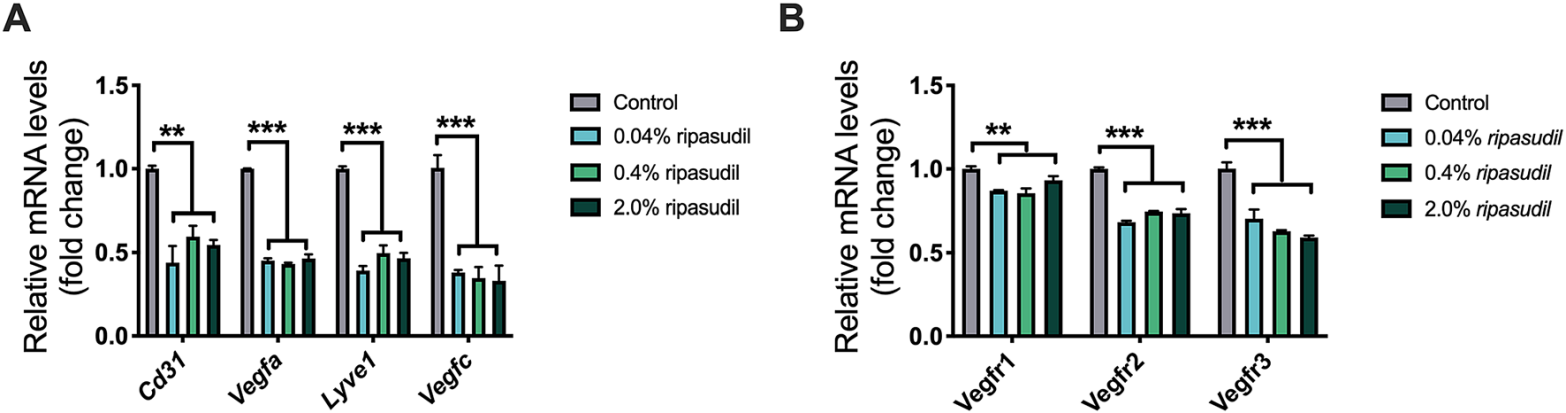
Expression of angiogenesis and lymphangiogenesis markers in the grafted cornea on day 14 post-transplantation. (**A**) mRNA levels of angiogenesis (*Cd31* and *Vegfa*) and lymphangiogenesis (*Lyve1* and *Vegfc*) markers in the 0.04%, 0.4%, and 2.0% ripasudil groups compared to those in the control (n = 3, one-way ANOVA; **p < 0.01, ***p < 0.001). (**B**) mRNA levels of *Vegfr1*, *2*, and *3* in the 0.04%, 0.4%, and 2.0% ripasudil groups compared to those in the control (n = 3, one-way ANOVA; **p < 0.01, ***p < 0.001). *Vegf* vascular endothelial growth factor, *Lyve1* lymphatic vessel endothelial hyaluronan receptor-1, *ANOVA* analysis of variance, *Vegfr* vascular endothelial growth factor receptor.

To investigate the effects of the topical administration of ripasudil on angiogenic signals in grafted corneas, we analyzed the mRNA levels of angiogenic and lymphangiogenic genes in the grafted cornea through reverse transcription-quantitative PCR (RT-qPCR) on day 14 post-transplantation (Fig. 2). Pecam1 (*Cd31*), *Vegfa*, *Vegfc*, *Lyve1*, and *Vegf receptor* (*Vegfr*)*1, 2, 3* mRNA were significantly downregulated in the ripasudil groups than in the control group (Fig. 2A,B; n = 3).

### Topical administration of ripasudil decreased leukocyte infiltration and inflammation-related mRNA expression in grafted corneas

**Figure 3:**
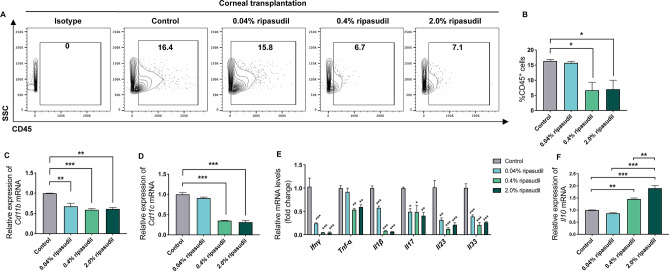
Leukocyte infiltration and inflammation-related mRNA levels in corneal grafts. (**A**) Representative flow cytometry plots and (**B**) statistical analysis of flow cytometry data showing the frequency of occurrence of infiltrating CD45^+^ leukocytes in grafted corneas at day 14 post-transplantation (n = 3, one-way ANOVA; *p < 0.05). mRNA levels of *Cd11b* (**C**) and *Cd11c* (**D**) expressed on the surface of leukocytes, including neutrophils, macrophages, and dendritic cells, in grafted corneas on day 14 post-transplantation (*Cd11b* n = 3, one-way ANOVA, **p < 0.01, ***p < 0.001; *Cd11c* n = 3, one-way ANOVA, ***p < 0.001). (**E**) mRNA levels of inflammation-related markers in grafted corneas on day 14 post-transplantation (n = 3, one-way ANOVA; *p < 0.05, **p < 0.01, ***p < 0.001). (**F**) mRNA level of Interleukin (*Il*)*10* in the grafted cornea on day 14 post-transplantation (n = 3, one-way ANOVA; **p < 0.01, ***p < 0.001). Statistical analyses of flow cytometry data were performed using FlowJo software X 10.5.3. (FlowJo LLC, Ashland, OR, USA; purchased from https://www.flowjo.com). *ANOVA* analysis of variance, *Ifnγ* Interferonγ, *Tnf-α* Tumor necrosis factor-α.

To determine the effect of ripasudil on the leukocyte infiltration of allografts, we examined CD45^+^ leukocytes in the grafted corneas through flow cytometry. CD45^+^ leukocytes in the grafted corneas were enumerated through flow cytometry on day 14 post-transplantation (Fig. 3A). The frequency of occurrence of CD45^+^ cells was significantly lower in the grafted corneas in the 0.4% and 2.0% ripasudil groups than in the control group (Fig. 3B, n = 3, control vs. 0.4% ripasudil, *p* = 0.031; control vs. 2.0% ripasudil, *p* = 0.039). The expression levels of *Cd11b* and *Cd11c*, surface markers of leukocytes such as neutrophils, macrophages, and dendritic cells (DCs)^21–23^, were significantly lower in the 0.4% and 2.0% ripasudil groups than in the control group (Fig. 3C, Fig. 3D, n = 3). The expression levels of inflammatory cytokines in the grafted corneas were assessed through RT-qPCR on day 14 post-transplantation. *Interferon* (*Ifn*)*γ, Tumor necrosis factor* (*Tnf*)-*α, Interleukin* (*Il*)*1β, Il17, Il23*, and *Il33* mRNA were significantly downregulated in the ripasudil groups than in the control group (Fig. 3E). Furthermore, the mRNA level of the immunoregulatory cytokine *Il10* was significantly higher in the grafted corneas in the 0.4% and 2.0% ripasudil groups than in the control group (Fig. 3F, n = 3, *p* < 0.01 and *p* < 0.001, respectively).

### Topical administration of ripasudil suppressed DC maturation, and IFNγ- and IL-17- expressing effector T cell generation in dLNs

**Figure 4:**
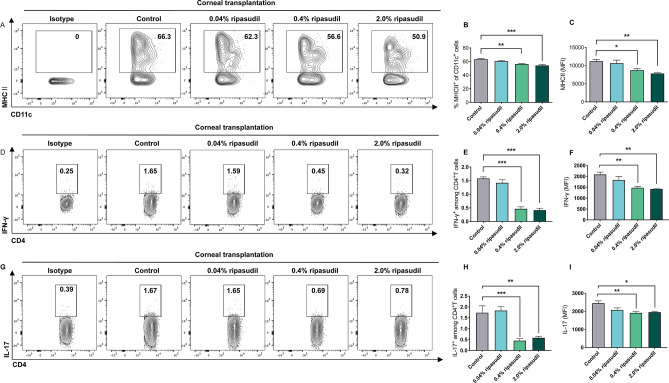
Topical administration of ripasudil suppressed dendritic cells (DCs) maturation, and Interferon (IFN)γ- and Interleukin (IL)-17-expressing effector T cell generation in draining lymph nodes (dLNs). (**A**) Representative flow cytometry plot showing major histocompatibility complex (MHC) II^+^ CD11c^+^ DCs in dLNs 14 days after corneal transplantation. (**B**) Frequencies of MHC II^+^ CD11c^+^ DCs in dLNs. (**C**) Mean fluorescence intensity of MHC II in CD11c^+^ cells from dLNs. (**D**) Representative flow cytometry plot showing CD4^+^ IFNγ^+^ cells. (**E**) Frequencies of occurrence of CD4^+^ IFNγ^+^ cells in dLNs (**F**) Mean fluorescence intensity of IFNγ in CD4^+^ cells from dLNs. (**G**) Representative flow cytometry plot showing CD4^+^ IL-17^+^ cells. (**H**) Frequencies of occurrence of CD4^+^ IL-17^+^ cells in dLNs. (**I**) Mean fluorescence intensity of IL-17 in CD4^+^ cells from dLNs. Statistical analyses of flow cytometry data were performed using FlowJo software X 10.5.3. (FlowJo LLC, Ashland, OR, USA; purchased from https://www.flowjo.com).

To investigate the effect of topical administration of ripasudil on dendritic cells (DCs) maturation, we determined the frequency of occurrence of CD11c^+^ major histocompatibility complex (MHC) II^+^ DCs and the expression of MHC II in the draining lymph nodes (dLNs) through flow cytometry 14 days after corneal transplantation (Fig. 4A–C). The frequency of occurrence of CD11c^+^ MHC II^+^ DCs was significantly lower in the 0.4% and 2.0% ripasudil groups than in the control group (Fig. 4B; n = 5; *p* = 0.002, *p* < 0.001, respectively). Furthermore, the mean fluorescence intensity of MHC II among dLNs was significantly lower in the 0.4% and 2.0% ripasudil groups than in the control group (Fig. 4C; n = 5; *p* = 0.029 and *p* = 0.002, respectively). The frequencies of IFNγ- and IL-17-expressing T cells (Fig. 4D,E,G,H) and mean fluorescence intensities of IFNγ and IL-17 (Fig. 4F; n = 5; *p* = 0.002, *p* = 0.001, respectively and 4I; n = 5; *p* = 0.007, *p* = 0.013, respectively) were lower in the 0.4% and 2.0% ripasudil groups than in the control.

### Topical administration of ripasudil promoted re-epithelization of the grafted cornea

**Figure 5:**
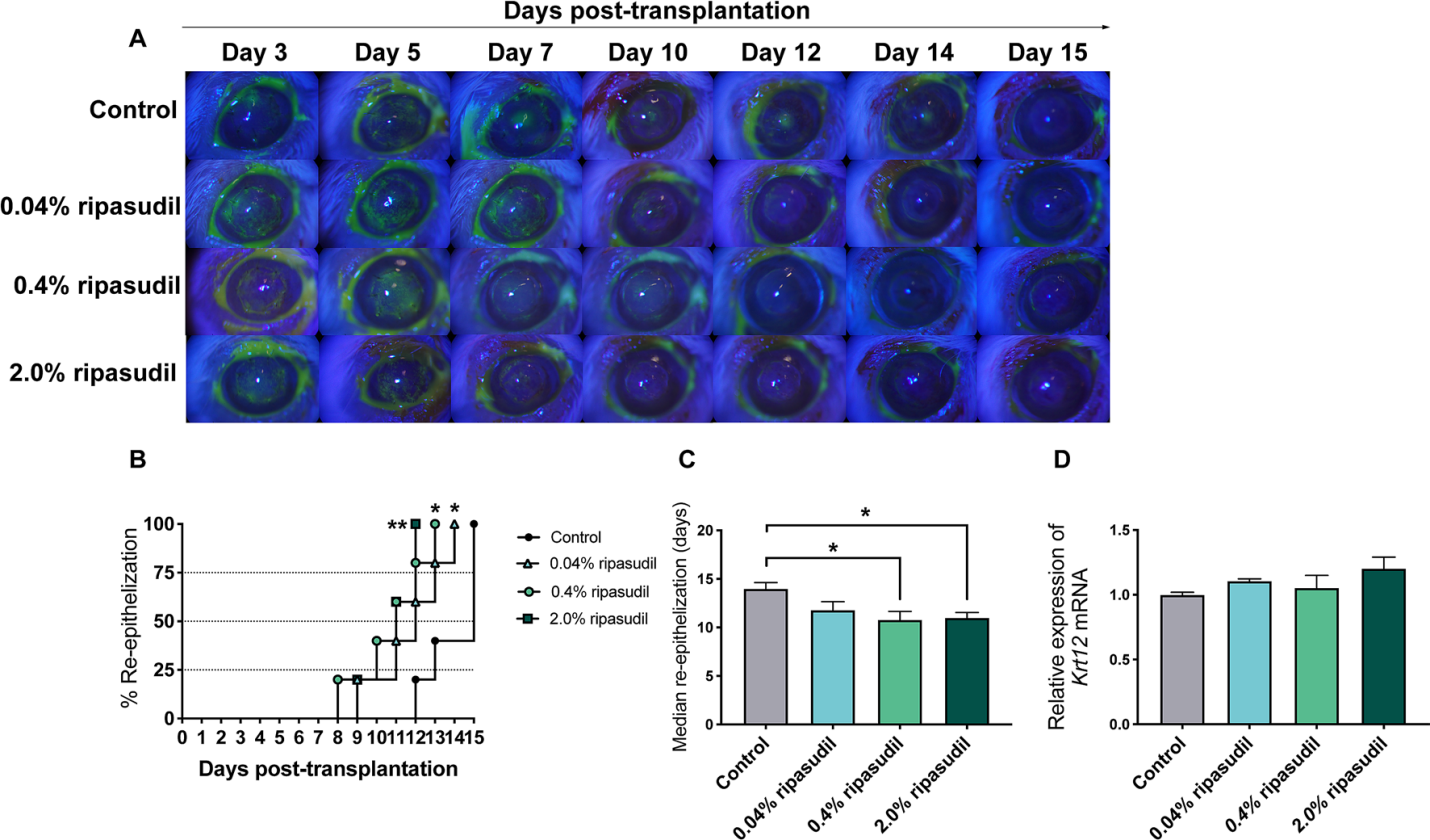
Ripasudil promoted the re-epithelization of corneal grafts. (**A**) Representative images of grafted corneas with fluorescein staining after topical administration with ripasudil post-corneal transplantation (n = 5 mice per group). (**B**) Re-epithelization rate of corneal grafts after treatment with 0.04%, 0.4%, and 2.0% ripasudil post-corneal transplantation (*p = 0.018, *p = 0.018, **p = 0.008, respectively; log-rank test, n = 5 mice per group). (**C**) Median time required for re-epithelization of corneal grafts after treatment with 0.4% and 2.0% ripasudil post-corneal transplantation (Mann–Whiteney test, *p = 0.040 and *p = 0.024, respectively). (**D**) Relative mRNA level of cytokeratin 12 (*Krt12*) did not differ significantly between the groups (one-way ANOVA, n = 5 mice per group). The re-epithelization rate of corneal grafts was analyzed using ImageJ version 1.53a (National Institutes of Health, Bethesda, MD, USA; available at https://rsb.info.nih.gov/ij/index.html)^57,58^. *ANOVA* analysis of variance.

Re-epithelization of a grafted cornea is essential not only for the potential immunological role of the corneal epithelium but also for corneal surface protection. Figure 5A shows representative images of re-epithelization of the grafted cornea obtained using a slit-lamp biomicroscope with cobalt blue light. Topical administration of ripasudil significantly promoted re-epithelization of the grafted cornea (Fig. 5B, n = 5; 0.04% ripasudil, *p* = 0.047; 0.4% ripasudil, *p* = 0.002; 2.0% ripasudil, *p* = 0.008). Topical administration of ripasudil improved the median re-epithelization time in the 0.4% and 2.0% ripasudil groups compared to that in the control group (Fig. 5C, *p* = 5; *p* = 0.103, *p* = 0.040, *p* = 0.024 for 0.04%, 0.4%, and 2.0% ripasudil vs. control, respectively). The expression of cytokeratin 12 (*Krt12*), a corneal epithelial differentiation marker^24^, did not differ significantly between the groups (Fig. 5D).

### Topical administration of ripasudil prolonged graft survival

**Figure 6:**
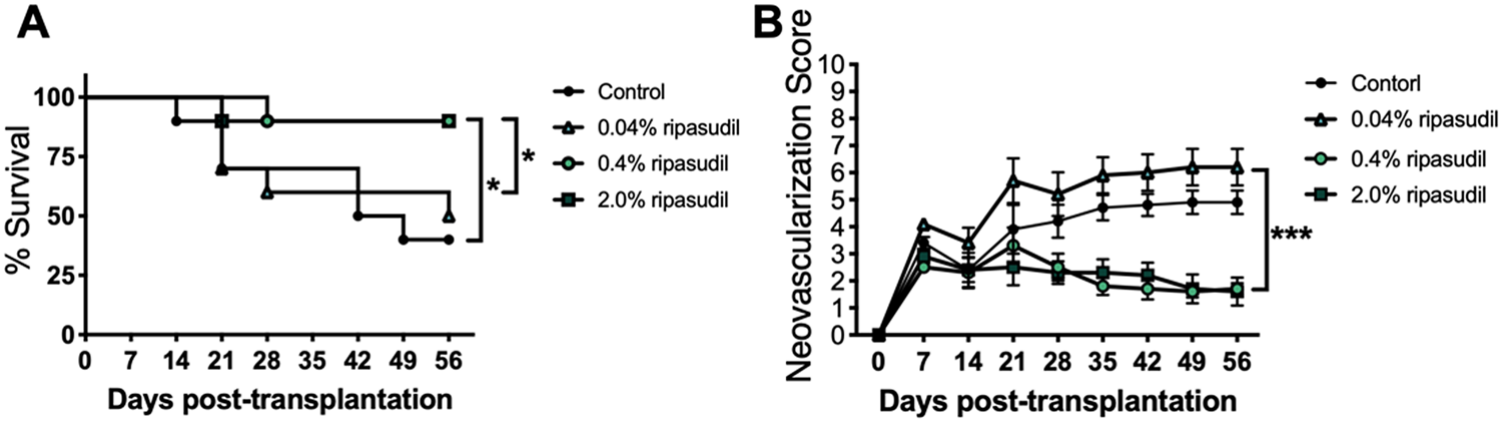
Topical administration of ripasudil promoted corneal graft survival. (**A**) Weekly examinations of grafted corneas for 8 weeks post-transplantation treated with 0.4% and 2.0% ripasudil (log-rank test, *p = 0.026 and *p = 0.025, respectively; n = 10 mice per group). (**B**) Weekly examinations of neovascularization scores of grafted corneas 8 weeks post-transplantation (two-way ANOVA, ***p < 0.001, ***p < 0.001, respectively, n = 10 mice per group). (**C**) Weekly examinations of graft opacity scores of grafted corneas 8 weeks post-transplantation (two-way ANOVA, ***p < 0.001, ***p < 0.001, respectively, n = 10 mice per group). *ANOVA* analysis of variance.

Administration of 0.4% and 2.0% ripasudil significantly prolonged graft survival (Fig. 6A, n = 7; 0.4% ripasudil, *p* = 0.024; 2.0% ripasudil, *p* = 0.025) and reduced neovascularization (Fig. 6B, n = 7; *p* < 0.001 for both) compared to that in the control group.

## Discussion

In this study, we investigated the efficacy of ripasudil, a ROCK inhibitor, in controlling the immune reactions and wound healing responses upon corneal transplantation when applied topically on a transplanted cornea. We found that topical administration of ripasudil mediates immunological and wound healing responses owing to its ability to be rapidly deactivated downstream of neovascularization and inflammation. These findings suggest that topical administration of ripasudil might be an effective treatment alternative for the management of corneal transplantation.

**Figure 7:**
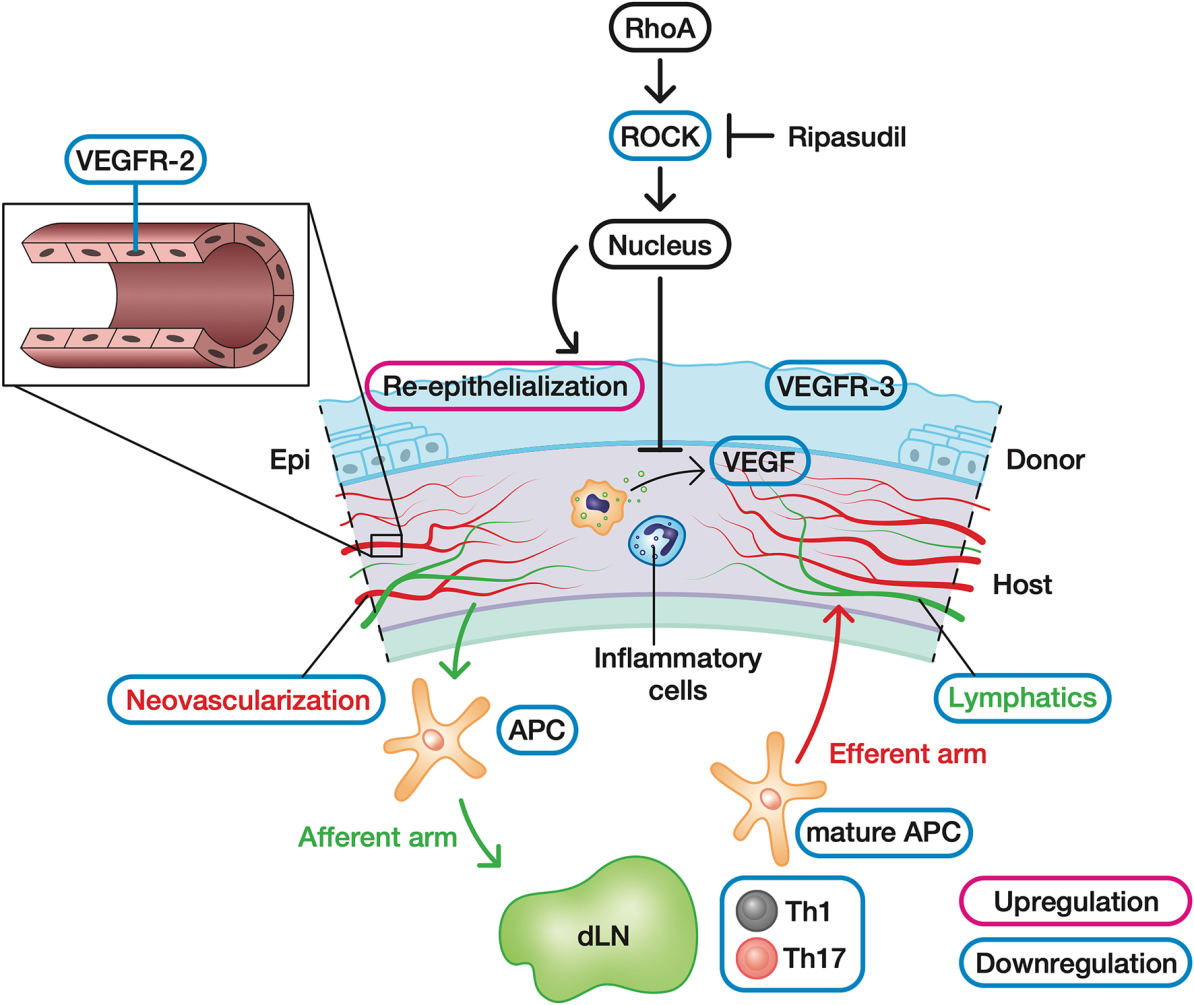
Mechanism of action of topically applied ripasudil involves suppression of local angiogenesis. Topical administration of the ROCK inhibitor ripasudil suppressed the local upregulation of proangiogenic factors and promoted graft re-epithelization, resulting in the reduction in circulating immune responses. *Rho-A* ras homolog family member A, *ROCK* Rho-associated coiled-coil-containing protein kinase, *VEGF* vascular endothelial growth factor, *VEGFR* vascular endothelial growth factor receptor, *APC* antigen presenting cell, *dLN* draining lymph node.

After corneal transplantation, upregulation of proangiogenic and proinflammatory factors, along with epithelial and stromal damage at the local graft site, cause corneal infiltration of immune cells and induction of neovascularization and lymphangiogenesis^25,26^. The circulating immune responses of afferent (lymphatic) and efferent (vascular) arms contribute to the loss of immune privilege and subsequent rejection^27^. These results suggest that topical administration of ripasudil locally suppressed the upregulation of proangiogenic and proinflammatory factors and promoted graft re-epithelization, resulting in the reduction of circulating immune responses (Fig. 7).

In corneal transplantation, induced local inflammation and epithelial and stromal damage lead to infiltration of inflammatory cells, primarily neutrophils and macrophages^28^. The infiltrating neutrophils and macrophages secrete VEGF. VEGFR-2 along with its ligands, VEGF-A and VEGF-C, is the principal signaling receptor for vascular endothelial cells. As ripasudil selectively inhibits the ROCK pathway and VEGF secretion in these cells, VEGF-A and VEGF-C secretion may have been reduced. We found that topical administration of ripasudil downregulated VEGFR-2 because the vascular endothelial cells themselves were reduced in the grafted cornea by the immunosuppressive function of ripasudil. Previous studies have reported that ROCK inhibitors regulate the proliferation of vascular endothelial cells by reducing VEGF-induced ROCK activation^20,29^. To determine the direct effects of ripasudil on vascular endothelial cells, further in vitro experiments are needed.

Angiogenesis is accompanied through significant inflammation^30^. After corneal transplantation, inflammatory cytokines are released, and inflammatory cells are recruited to the grafted cornea^25,31^. Thereafter, CNV is induced in the grafted cornea, delaying the epithelialization of corneal grafts, and triggering corneal rejection^32^. Topical administration of ripasudil reduced the number of CD45^+^ infiltrating cells and inflammatory cytokine expression in the grafted cornea. TNF-α and IL-1β were significantly downregulated in the 0.4% and 2.0% ripasudil groups compared to the 0.04% ripasudil group, indicating that their downregulation contributes to corneal graft survival. In particular, of the cytokines examined in this study, only TNF-α was not suppressed upon 0.04% ripasudil administration in corneal grafts, suggesting that TNF-α is a potential key regulator of suppressing inflammation, angiogenesis, and lymphangiogenesis upon corneal transplantation^33,34^. A previous study reported that ROCK inhibition protected the vascular endothelium by inhibiting neutrophil adhesion^35^ and downregulating inflammatory cytokines including VEGF, TNF-α, matrix metalloproteinase (MMP)-2, and MMP-9^36–38^. Furthermore, this study elucidates the efficacy of ripasudil in reducing the infiltration of inflammatory cells to sites in the grafted cornea after reducing the levels of the associated inflammatory cytokines at the site of injury.

Delayed corneal epithelial wound healing causes corneal infiltration of inflammatory cells and subsequent VEGF secretion^39,40^. Therefore, epithelial regeneration after corneal transplantation is important for graft survival. ROCK contributes to cell proliferation and migration^14^. The migration of recipient corneal epithelial cells is initiated 5 days after transplantation. Subsequently, the recipient epithelial cells disperse and are distributed in the graft cornea from the periphery to the center by approximately 10 days post-transplantation. Finally, re-epithelization of the grafted cornea is completed 15 days after transplantation^41^. Administration of 0.4% and 2.0% ripasudil promoted re-epithelization of the grafted cornea, concurrent with the a previous report on would healing^42^. As the epithelium serves as a significant barrier to graft acceptance^41^, rapid graft re-epithelization is critical for visual acuity, graft transparency, and protection of the stroma against infection and melting^43^. In particular, promotion of re-epithelization by the ROCK inhibitor may suppress the induction of inflammation, CNV, infection, and even graft rejection.

Owing to the reduction in local angiogenic factor levels and inflammation and promotion of epithelial regeneration by ripasudil administration, angiogenesis and lymphangiogenesis were suppressed, and graft survival was prolonged. DCs and Th1 and Th17 cells act as important mediators in the immune response at the early stage of corneal allograft rejection^8,44–46^. We found that the numbers of mature DCs and IFNγ^+^ Th1 and IL-17^+^ Th17 cells decreased in the dLNs, indicating that ripasudil administration inhibited angiogenesis and lymphangiogenesis and subsequently suppressed the reduction in antigen presentation in the dLNs and migration of the Th1 and Th17 cells to the grafted cornea. Furthermore, fasudil, a ROCK inhibitor, regulates the proportions of IFNγ^+^ Th1 and IL-17^+^ Th17 cells, indicating that the ROCK inhibitor itself may have a local immunosuppressive effect^47^. In the efferent arm, anti-neovascularization treatments are especially important for blocking the direct and indirect migration of donor antigen-presenting cells^48^. The afferent arm is characterized by migration of the allogeneic antigens via the lymphatic tissues. This study shows that topical administration of ripasudil suppressed lymphangiogenesis by downregulating VEGFR-3 along with its ligands, VEGF-C and VEGF-D. A previous study reported the interaction between angiogenesis and lymphangiogenesis^49^, suggesting that the anti-angiogenic property of ripasudil was responsible for suppressing angiogenesis and lymphangiogenesis in the grafted cornea.

There are several limitations to this study. This study identified the inhibitory effects of ripasudil for CNV using a corneal transplantation model. However, since CNV occurs in various diseases, further studies using different CNV models are necessary. Furthermore, this study did not investigate the potential immunosuppressive effect of topical administration of ripasudil with steroids. Steroids are frequently topically administered after corneal transplantation^50^. Therefore, it is necessary to investigate whether adding ripasudil to the steroid after corneal transplantation will further suppress the rejection. Recent studies have reported a protective effect of ROCK inhibitors on corneal endothelial cells^51,52^. Although the corneal endothelial cell density in corneal transplantation was not investigated in this study, we speculate that ripasudil may be effective for the long-term survival of corneal grafts because of the protective effect of ROCK inhibitors on corneal endothelial cells.

In summary, the ROCK inhibitor ripasudil inhibited CNV and inflammation and subsequently promoted graft survival. Ripasudil administration might satisfy unmet medical needs in the treatment of corneal transplantation, which require suppression of corneal neovascularization and inflammation.

## Materials and methods

### Animals and anesthesia

BALB/c (H-2d) and C57BL/6 (H-2b) male mice (6–8 weeks old) were purchased from Sankyo Labo Service Corporation, Inc. (Tokyo, Japan). All animal experiments were approved by the Institutional Animal Care and Use Committee of the Juntendo University Graduate School of Medicine (Approval No. 2020231) and were conducted in accordance with the Association for Research in Vision and Ophthalmology statement for the Use of Animals in Ophthalmic and Vision Research. Anesthesia was administered intraperitoneally (ketamine/xylazine solution at 120 mg/kg body weight and 20 mg/kg body weight, respectively).

### Allogeneic corneal transplantation

For allogeneic corneal transplantation, the corneas of C57BL/6 mice were grafted onto BALB/c host beds as described previously^53^. In brief, the central cornea (2 mm in diameter) was excised from a donor C57BL/6 mouse using scissors (Vannas-Storz Instruments, San Dimas, CA, USA). The graft bed was prepared by excising a 1.5-mm site in the central cornea of a BALB/c mouse. The donor button was then placed onto the recipient bed and secured with eight interrupted 11-0 nylon sutures. After surgery, the host eyelids were closed for 3 days, and the interrupted corneal sutures were removed 7 days after surgery.

### Grafted cornea assessment

The graft’s neovascularization score, opacity score, and survival rate were evaluated for 8 weeks using a slit-lamp biomicroscope. We used a standardized scoring system to assess the neovascularization score (range, 0–8) and opacity score (range, 0–5+)^53^. Corneas with an opacity score of 2+ for two consecutive examinations were rejected. Re-epithelization of the grafted cornea was assessed using 0.5% fluorescein staining under a slit-lamp biomicroscope with cobalt blue light.

### Eye drop treatment

Ripasudil (K-115), a novel ROCK inhibitor, was obtained from Kowa Company Ltd. (Nagoya, Japan)^18^. To assess the pharmacological efficacy of ripasudil in the murine cornea transplantation model, 0.04%, 0.4%, and 2.0% ripasudil (n = 5–7 mice/group) was administered thrice daily in accordance with a previous study^20,54,55^. The vehicle of ripasudil was used as the control^56^, which contained sodium dihydrogen phosphate as a buffering agent, glycerin as an isotonic agent, sodium hydroxide as a pH-adjusting agent (pH range 5–7), and benzalkonium chloride as a preservative.

### Corneal whole mount and immunofluorescence staining

Freshly excised corneas were washed with phosphate-buffered saline on day 14 post-transplantation. The corneal epithelium was removed after incubation with 20 mM ethylenediaminetetraacetic acid for 60 min at 37 °C, fixed in acetone for 15 min at 20–22 °C, and blocked in 2% bovine serum albumin for 60 min. The corneas were double-stained overnight for CD31 (Santa Cruz Biotechnology, Dallas, TX, USA) and LYVE-1 (AF2125, R&D Systems, MN, USA) using goat anti-mouse fluorescein isothiocyanate (FITC)-conjugated CD31 (1:100) and purified goat anti-mouse (1:400) LYVE-1, respectively, as described previously^57^. Cy3-conjugated donkey anti-goat (1:2000, Jackson ImmunoResearch Laboratories, West Grove, PA, USA) antibody was then added as a secondary antibody and incubated for 2 h. Stained whole corneas were mounted in Vectashield with 4′,6-diamidino-2-phenylindole (DAPI) (Vector Laboratories Inc., Burlingame, CA, USA). The stained whole mount corneas were observed under a fluorescence microscope (BZ-X710, Keyence, Osaka, Japan). The area of the whole grafted cornea covered by blood or lymphatic vessels was analyzed using ImageJ version 1.53a (National Institutes of Health, Bethesda, MD, USA; available at https://rsb.info.nih.gov/ij/index.html)^57,58^.

### RNA isolation and reverse transcription-quantitative PCR (RT-qPCR)

The excised corneas were immediately submerged in RNAlater solution (Ambion, Austin, TX, USA). Total RNA was isolated from five corneas per group using a NucleoSpin RNA isolation kit (Macherey-Nagal GmbH, Duren, Germany) in accordance with the manufacturer’s instructions. The cDNA was reverse-transcribed from total RNA using random primers and the ReverTra Ace qPCR RT kit (Toyobo, Osaka, Japan) in accordance with the manufacturer’s guidelines. The qPCR primers specific for mouse mRNA are enlisted in Table S1. qPCR was performed with the ABI PRISM 7300 HT sequence detection system using the FAST-SYBR Green master mix (Life Technology Japan, Tokyo, Japan). Results were analyzed using the comparative cycle threshold method, and *Gapdh* mRNA expression in the same cDNA was used as the internal control.

### Flow cytometry analysis

Corneas and ipsilateral dLNs were harvested, and single-cell suspensions were prepared as described previously^53,59^. To avoid non-specific staining, cells were blocked with an anti-FcR blocking antibody (eBioscience, San Diego, CA, USA). The isolated cells were stained with the respective antibodies. Mature DCs were stained with anti-CD11c Alexa488 (N418, BioLegend, CA, USA), anti-CD45 PE (30-F11, eBioscience), and anti-I-A/I-E PeCy7 (M5/114.15.2, BioLegend). For intracellular IFNγ and IL-17 staining, the cells were stimulated with 50 ng/mL phorbol 12-myristate 13-acetate, and 500 ng/mL ionomycin (Sigma-Aldrich, St. Louis, MO, USA) for 6 h at 37 °C in a 5% CO_2_ incubator in the presence of GolgiStop (0.7 μL per 100 μL cell culture; BD Biosciences, San Jose, CA, USA) to inhibit cytokine secretion. The cells were then stained with anti-CD4 FITC, anti-IFNγ APC (XMG1.2), and anti-IL-17 PECy7 (TC11-18H10.1) (BioLegend) antibodies. All antibodies and their matched isotype controls, and the fixation and permeabilization buffers were purchased from eBioscience. The stained cells were examined using LSR Fortessa (BD Biosciences, Franklin Lakes, NJ, USA) and analyzed using FlowJo software X 10.5.3. (FlowJo LLC, Ashland, OR, USA; purchased from https://www.flowjo.com).

### Statistical analysis

Experiments with more than two groups were analyzed using one-way or two-way analysis of variance (ANOVA) with Bonferroni’s multiple comparison post-hoc test. The Mann–Whitney test was performed to compare medians between the groups. Kaplan–Meier analysis was performed to construct corneal graft re-epithelization curves and to evaluate graft survival post-corneal transplantation, and the log-rank test was performed to compare corneal re-epithelization of the grafted corneas and graft survival post-corneal transplantation. Data are presented as mean ± standard error of mean values and were considered statistically significant at *p* < 0.05. All statistical analyses were performed using Prism version 8.0 software (GraphPad, La Jolla, CA, USA).

## Supplementary information

Supplementary Table S1.

## Data Availability

All data generated or analyzed during this study are included in this published article (and its Supplementary Information files).
