# Supplementary material for: Novel immunotherapeutic effects of topically administered ripasudil (K-115) on corneal allograft survival
Source: Sci Rep. 2020 Nov 13;10:19817. doi: 10.1038/s41598-020-76882-w (PMC7666179; doi:10.1038/s41598-020-76882-w)
Supplement: Supplementary file 1 — Supplementary Table S1. [file 41598_2020_76882_MOESM1_ESM.pdf]

Novel Immunotherapeutic Effects of Topically Administered Ripasudil (K-115) on Corneal Allograft Survival

Takenori Inomata, MD, PhD[1,2,3,4,5*], Keiichi Fujimoto, MD[1,2,5], Yuichi Okumura, MD[1,2,3,5], Jun Zhu, MD[1], Kenta Fujio, MD[1,2,5], Hurramhon Shokirova, MD[1], Maria Miura, MD[1,5], Mikiko Okano, MD[1], Toshinari Funaki, MD, PhD[1,6], Jaemyoung Sung, MS[1,7], Naoko Negishi, PhD[8,9], and Akira Murakami, MD, PhD[1,2,5]

Table S1. Primer sets for real-time PCR.

Gene	Forward (5′ - 3′)	Reverse (5′ - 3′)
GAPDH	AAGGGCTCATGACCACAGTC	GGATGACCTTGCCCACAG
CD11b	CGGAAGGATTCAGCAAGCCAGAAC	AGCTGGACTCAGCAGGCTTTAC
CD11c	TTGGGTGCCCATAGAGCTGAAG	CATTGGGTGAGTGGGTTCTGAG
IFNγ	CGGCACAGTCATTGAAAGCC	TGTCACCATCCTTTTGCCAGT
IL-10	AGGCGCTGTCATCGATTTCT	TGGCCTTGTAGACACCTTGG
IL-1β	TGCCACCTTTTGACAGTGATG	ATGTGCTGCTGCGAGATTTG
IL-17A	GCAGGAGAATTGCATCCTGGTGTG	AGGACACAAGACGTGCTTCCAG
IL-23A	GACTCAAGGACAACAGCCAGTTC	TCCTAGTAGGGAGGTGTGAAGTTG
IL-33	TCCAACTCCAAGATTTCCCCG	CATGCAGTAGACATGGCAGAA
TNF-α	AGCCCACGTCGTAGCAAAC	TTTGAGATCCATGCCGTTGG
Cytokeratin12	CGCTGGGTCTCAGAGTGATT	CTGACTCTGGCAGAAACGATCTTA
LYVE-1	TGGGAAGAATGGCAAAGGTGTCC	ATGCAGGAGTTAACCCAGGTGTCG
Pecam-1	TTGAGCCTCACCAAGAGAACGG	ACTCTCGCAATCCAGGAATCGG
VEGF-A	TGTACCTCCACCATGCCAAGTG	TGGGACTTCTGCTCTCCTTCTGTC
VEGF-C	TCCTGGGAAATGTGCCTGTGAATG	GCACACGGTCTTCTGTAACAACTG
VEGFR-1	GAGGAGGATGAGGGTGTCTATAGGT	GTGATCAGCTCCAGGTTTGACTT

VEGFR-2	GCCCTGCCTGTGGTCTCACTAC	CAAAGCATTGCCCATTCGAT
VEGFR-3	GTGCTCAAAGAGGTGACCGA	TGAGGAGGCACATTCACCAC

IFN; interferon, IL; interleukin, TNF; tumor necrosis factor, LYVE; lymphatic vessel endothelial hyaluronan receptor, VEGF; vascular endothelial growth factor, VEGFR; vascular endothelial growth factor receptor.
